# Marine sponge derived natural products as inhibitors of mycothiol-S-conjugate amidase

**DOI:** 10.6026/97320630013256

**Published:** 2017-08-31

**Authors:** Akansha Saxena, Sanjay Mishra

**Affiliations:** 1 School of Biotechnology, IFTM University, Lodhipur Rajput, Delhi Road, Moradabad 244001, U.P., India

**Keywords:** Mycothiol-S-conjugate amidase, Mycobacterium tuberculosis, Mca, NAMD, VMD

## Abstract

Marine sponges have potential sources for secondary metabolites and are considered as a drug treasure house. In this work, 3D model of Mycothiol-S-conjugate amidase (Mca) was determined by comparative homology modeling program MODELLER based on the crystal structure of 1-D-myo-inositol 2-acetamido-2-deoxy-alpha-D-glucopyranoside deacetylase (MshB) from Mycobacterium tuberculosis as a template. The computed model's energy was minimized and validated to obtain a stable model structure. Stable model was used for docking of nineteen bioactive compounds isolated from marine sponges against Mca using AutoDock 4.2. The docked complexes were validated and enumerated based on the AutoDock Scoring function to pick out the best marine inhibitors based on binding energy. Thus from the entire marine compounds which were docked, we got best one (Arenosclerin E) of them with optimal binding energy -13.11 kcal/mol. Further the best-docked complex was analyzed through Python Molecular Viewer software for their interaction studies. The docked protein - inhibitor complex structure was optimized using molecular dynamics simulation for 5 ps with the CHARMM-22 force field using NAMD incorporated in VMD 1.9.2 and then evaluating the stability of complex structure by calculating RMSD values. Thus from the Complex scoring and binding ability its deciphered that this marine derived compound could be promising inhibitor for Mca as drug target yet pharmacological studies have to confirm it.

## Background

Tuberculosis (TB) is one of the most deadly infectious diseases in humans caused by Mycobacterium tuberculosis. TB, with AIDS, is the leading infectious cause of adult mortality in the world, causing between 1.5 and 2 million deaths per year and infected almost one-third of the world's population [1]. The majority of people afflicted with TB live in developing countries. WHO in March 2017 estimated that six countries account for 60% of the total, with India leading the count, followed by Indonesia, China, Nigeria, Pakistan and South Africa [2]. Globally in 2015, an estimated 4.8 lack people developed multidrug-resistant TB [2]. M. tuberculosis infection is cured by chemotherapy, although the treatment takes 6-9 months [3]. Currently, TB chemotherapy is made up of a cocktail of four antibiotics isoniazid, rifampin, pyrazinamide and ethambutol given to patient for six months [3]. If the treatment fails due to bacterial drug resistance, second-line drugs will given to patients, such as para-aminosalicylate (PAS), fluoroquinolones, kanamycin, ethionamide, cycloserine and capreomycin that had more toxic with serious side effects [3]. The growing problem of MDR-TB and the lack of drugs that effectively target persistent bacteria, stress the urgent need for identification of new antimicrobial targets.

Sulfur is an essential element for life and plays a central role in numerous microbial metabolic processes [4]. In its reduced form, sulfur is used in the biosynthesis of the amino acids cysteine and methionine. Cysteine is incorporated into biomolecules such as proteins, coenzymes, and mycothiol. Mycothiol regulates cellular redox status and is essential for M.tuberculosis survival [5]. Mycobacterial sulfur metabolism represents a promising new area for anti-TB therapy [6]. Most microbial sulfur metabolic pathways are absent in humans and therefore, represent unique targets for therapeutic intervention. Mycothiol (MSH) or 1D-myoinosityl 2-(N-acetyl-L-cysteinyl) amido-2-deoxy-α- Dglucopyranoside, is an unusual conjugate of N-acetylcysteine (AcCys) with 1D-myo-inosityl 2-acetamido-2-deoxy-α-Dglucopyranoside (GlcNAc-Ins), and is the major low-molecular mass thiol in most action-mycetes, including mycobacteria [7]. MSH is the functional equivalent of glutathione (GSH) in mycobacteria [6,8] and is associated with the protection of M. tuberculosis from toxic oxidants and antibiotics [9]. Two other important enzymes involved in MSH metabolism and detoxification are mycothione reductase (Mtr) and Mycothiol-Sconjugate Amidase (Mca). In these two Mca plays a critical role in mycobacterial detoxification of antibiotics [10]. Therefore, inhibitors of Mca could enhance the sensitivity of MSHproducing bacteria to antibiotics, establishing Mca as a promising new drug target.

The discovery of marine natural products has accelerated over the last two decades with the number of new compounds discovered annually increasing from 20 to more than 200 [11]. Natural products have interesting biomedical potential, pharmaceutical relevance and diverse biotechnological applications [12, 13, 14, 15, 16, 17]. Marine sponge crude extracts present a high incidence of antibacterial activity against terrestrial pathogenic bacteria [18, 19, 20, 21, 22, 23], but a low incidence of antibacterial activity against marine bacteria [18, 19, 24]. Moreover, sponge-derived antifouling molecules have been found to be less toxic, environmentally friendly biocides that are often very effective [25].

## Methodology

The amino acid sequences of Mycothiol-S-conjugate Amidase (Entry No: P9WJN1) from Mycobacterium tuberculosis (strain ATCC 25618/H37Rv) were retrieved from uniprot (http://www.uniprot.org/). The template of Mycothiol-Sconjugate Amidase (Mca) was downloaded from protein Databank (www.rcsb.org/pdb) with PDB ID 1Q74.

The template and target sequence was aligned using the align2d script available in MODELLER 9v18 [26]. Based on the alignment, five comparative models of the target sequence were built by MODELLER. The best model can be selected by picking the model with the lowest value of the Modeller objective function and DOPE score from a collection of models built by MODELLER. PROCHECK [27] was used to check the Stereochemical qualities of the model. Homology model of Mca protein was constructed using program Modeller9v18. After aligning target Mca with template 1Q74-A was used as input in Modeller program and five comparative models were generated. The model of Mca was validated with the help of Modeller objective function and DOPE score, which are the statistical parameter for the assessment of model using the standard Modeller energy function. The validated model was chosen for further studies and refinement.

The newly built homology models often produce unfavorable atomic distances, bond angles, Vander Waals radius overlapping and undesirable torsion angles. Therefore, it was essential to minimize the energy to regularize local bond and angle geometry as well as to relax close contacts in geometric chain. Models of Mca protein were optimized with the Variable Target Function Method (VTFM) with Conjugate Gradients (CG). Further using the Molecular Dynamics (MD) with Simulated Annealing (SA) method in Modeller program refined it. Among the above models, the most acceptable model was finalized by Ramachandran plot, which provides the residue position in particular segments based on phi (φ) and psi (ψ) angles between N-Cα and Cα-C atoms of residue. After the optimization procedure, the stereochemical qualities of the model is checked by PROCHECK [27]

Marine sponges are among the richest sources of pharmacologically active chemicals from marine organisms. As infectious microorganisms evolve and develop resistance to existing pharmaceuticals, the marine sponge provides potent leads against bacterial, viral, fungal and parasitic diseases. We have retrieve antibacterial compounds such as (S)-(+)- curcuphenol, Agelasine D, Arenosclerin E, Axinellamine B, Corallidictyal A, Cribrostatin 3, Cribrostatin 6, Cyclostellettamine A, Deoxytopsentin, Hamacanthin A, Ingenamine G, Isojaspic acid, Cacospongin D, Jaspaquinol, Latrunculin B, Melophlin C, Petrosamine B, Psammaplin A derived from marine sponges from literature [28]. The 3D structures of known 19 inhibitors were downloaded in .sdf format from pubchem compound database. They were later converted in .pdb format with the help of open babel [29] tool.

Docking of ninteen antibacterial isolated from marine sponges [28] against Mycothiol-S-conjugate Amidase (Mca) structure was done using molecular docking program AutoDock [30]. Gasteiger charges are added to the ligand and maximum 6 numbers of active torsions are given to the lead compounds using AutoDock tool [31]. Kollman charges and the solvation term were added to the protein structure. The Lamarckian genetic algorithm implemented in Autodock was used for docking.

## Results and discussion

Five models of Mca protein were generated by MODELLER using crystal structure of 1-D-myo-inositol 2-acetamido-2-deoxyalpha- D-glucopyranoside deacetylase (MshB) from Mycobacterium tuberculosis as a template for homology modeling and had 42% sequence identity with Mca protein. Among the five models best model was selected by picking the model with the lowest value of the MODELLER objective function and the DOPE score, which are reported at the end of the log file. In this work, the fourth model had the lowest objective function (1989.20532) and DOPE score (-27173.13086) is selected. Ramachandran plot drawn through PROCHECK [27] program validated the structural model with 90.7% of the total residues in most favoured region and residues in additional allowed regions was 6.9% and 1.2% in the generously allowed region. This stipulates that protein backbone dihedral angles phi (φ) and psi (ψ) occupied reasonably accurate positions in the selected 3D model. Only three residues were located in the disallowed region, which constituted 1.2% of the total protein.

**Figure 1:**
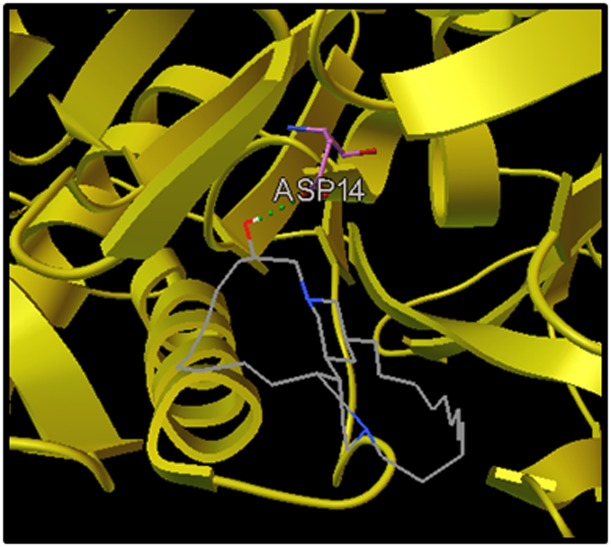
Docking orientation of Arenosclerin E with Mca protein. Complex depicting compound formed one H-bond with ASP14 of protein. Arenosclerin E is represented as lines and colored as atom type.

Docking studies predicted the interaction of ligands with protein and residues involved in this complex. For such interaction studies, the most important requirement was the proper orientation and conformation of ligand which fitted to the enzyme binding site appropriately and formed protein-ligand complex. Therefore, optimal interactions and the best AutoDock score were used as criteria to interpret the best conformation among the 10 conformations, generated by AutoDock program. The docking results of 19 compounds with Mca model were shown in Table 1. Among the above docked compounds Arenosclerin E had the lowest binding energy -13.11kcal/mol with Mca protein. Docking poses of the best conformation of Arenosclerin E in the binding site of modeled Mca protein was shown in Figure 1.

**Figure 2:**
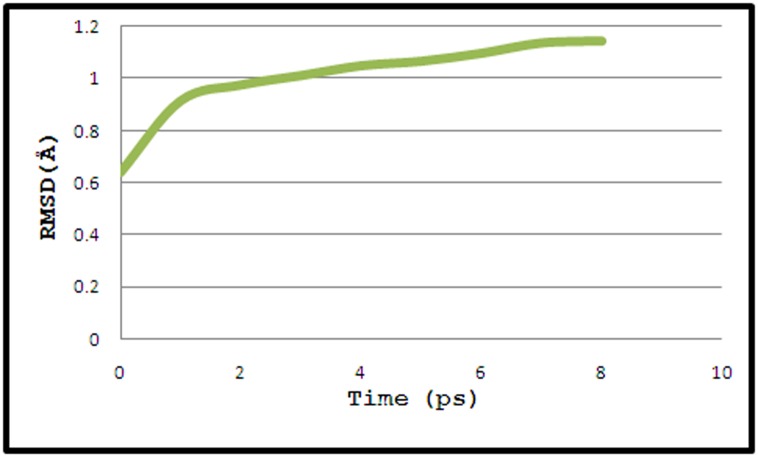
Graph displaying root mean square deviation (RMSD) of the backbone atoms of docked complex (Arenosclerin E - Mca protein) versus time at 310 K, resulted in highest peak at 1.14 Å.

Molecular dynamics simulations were done using the NAMD graphical interface module [32] incorporated in VMD 1.9.2 [33]. The protein-ligand complex was immersed in the center of a 50Å box of water molecules where all water molecule atoms were closer than 1.5 Å and a CHARMM22 parameter file for proteins and lipids was used in the force field for complexes. The psf was created from the initial pdb and topology files using psfgen package of VMD. After running psfgen, two new files were generated protein pdb and protein psf and by accessing PSF and PDB files; NAMD generated the trajectory DCD file. After the simulations, the results were analyzed in VMD by calculating the Root mean square deviation (RMSD) of the complex using rmsd tcl source file from the Tk console and finally rmsd.dat was saved and accessed in Microsoft office excel. RSMD, a crucial parameter to analyze the equilibration of MD trajectories, is estimated for backbone atoms of the Arenosclerin E - Mca protein complex (shown in Figure 2). Measurements of the backbone RMSD for the complex provided insights into the conformational stability.

## Conclusion

Homology Model built for Mca protein of Mycobacterium tuberculosis had high reliability and docking analysis showed that Arenosclerin E is a potent drug candidate for tuberculosis. Yet pharmacological studies have to confirm it.

## Figures and Tables

**Table 1:** Docking result of compounds with Mca.

Sl. No.	PubChem CID	Inhibitor	BE	IME	IE	TorE	VdwE	EE
1	156118	S)-(+)-curcuphenol	-6.54	-8.03	-0.74	1.49	-7.98	-0.05
2	11775482	Agelasine D	-7.46	-9.25	-1.94	1.79	-9.02	-0.23
3	44421344	Arenosclerin E	-13.11	-13.41	0.13	0.3	-10.26	-3.14
4	100962376	Axinellamine B	-9.02	-12	-2.65	2.98	-8.43	-3.57
6	190954	Corallidictyal A	-7.35	-7.94	-0.55	0.6	-7.86	-0.08
7	9926342	Cribrostatin 3	-5.72	-7.21	-1.21	1.49	-7.18	-0.04
8	9903535	Cribrostatin 6	-6.41	-7	-0.27	0.6	-6.96	-0.05
9	44422969	Cyclostellettamine A	-10.7	-10.7	0	0	-10.67	-0.08
10	183527	Deoxytopsentin	-7.63	-8.52	-0.97	0.89	-8.44	-0.08
11	3037568	Hamacanthin A	-7.34	-7.94	-0.53	0.6	-7.9	-0.04
12	101358565	Ingenamine G	-13.02	-13.32	0.19	0.3	-10.89	-2.42
13	46907602	Isojaspic acid	-7.02	-9.41	-1.39	2.39	-9.84	0.42
14	10251028	Cacospongin D	-4.03	-7.31	-1.75	3.28	-7.96	0.65
15	10110469	Jaspaquinol	-6.99	-9.98	-1.38	2.98	-9.69	-0.28
16	6436219	Latrunculin B	-7.56	-8.16	-1.67	0.6	-8.26	0.11
17	54713485	Melophlin C	-5.12	-8.7	-2.4	3.58	-8.76	0.06
18	21778132	Petrosamine B	-9.1	-9.1	0	0	-8.07	-1.03
19	6400741	Psammaplin A	-7.17	-12.24	-1.82	5.07	-11.56	-0.68

BE: Binding Energy; IME: Intermolecular Energy;IE: Internal Energy; TorE: Torsional Energy; VdwE:Vdw-lbDesolv Energy; EE: Electrostatic Energy.
